# Comparison of different interposition techniques after surgical resection of tarsal coalitions in children: a systematic review

**DOI:** 10.1097/BPB.0000000000001177

**Published:** 2024-04-09

**Authors:** Anne J. Spaans, Susanne E. Korbee, Nathalie C. Simoens, Christiaan J.A. van Bergen

**Affiliations:** a Department of Orthopaedic Surgery, Sint Maartenskliniek, Nijmegen/Boxmeer; b Department of Emergency Medicine, Rijnstate Hospital, Arnhem; c Department of Orthopaedic Surgery, Amphia Hospital, Breda; d Department of Orthopaedic Surgery and Sports Medicine, Erasmus University Medical Center – Sophia Children’s Hospital, Rotterdam, the Netherlands

**Keywords:** bone wax, calcaneonavicular, fat, foot deformities, surgery, talocalcaneal, tarsal coalition, tendons

## Abstract

In the surgical treatment of tarsal coalitions, it is unclear whether interposition material should be used to prevent recurrence. The aim of this review was to systematically examine the results of different interposition tissues after surgical resection of tarsal coalitions in children. A systematic review was performed according to the Preferred Reporting Items for Systematic Reviews and Meta-Analyses guidelines. Two independent investigators systematically searched electronic databases (PubMed, Embase, Cochrane) and included original articles reporting outcomes of tarsal coalition resection. The quality of included studies was assessed using the Methodological Index for Non-Randomized Studies (MINORS) criteria. Out of 294 articles, 21 studies examining 436 patients (581 feet), were included. The mean age was 12.2 years (range 7–18). There were 153 talocalcaneal, 425 calcaneonavicular, 2 naviculocuboidal, and 1 naviculocuneiform coalitions. The mean follow-up time was 58 months (range 12–276). In 96 feet, solely resection was performed. Resection and interposition were performed with muscle/tendon (n = 178), fat graft (n = 176), other material (n = 36), or a combination of interposition techniques (n = 95). Eighteen studies reported on recurrence, which was found in 45 of 485 feet (9%). The highest recurrence (17%) was described after muscle/tendon interposition for calcaneonavicular coalitions. However, a statistical comparison could not be performed. The included studies were diverse and the scientific quality was generally low (MINORS mean 7, range 3–20). Coalition resection with various interposition techniques results in low recurrence rates. It is unclear which interposition material shows the best results.

## Introduction

**Fig. 1:**
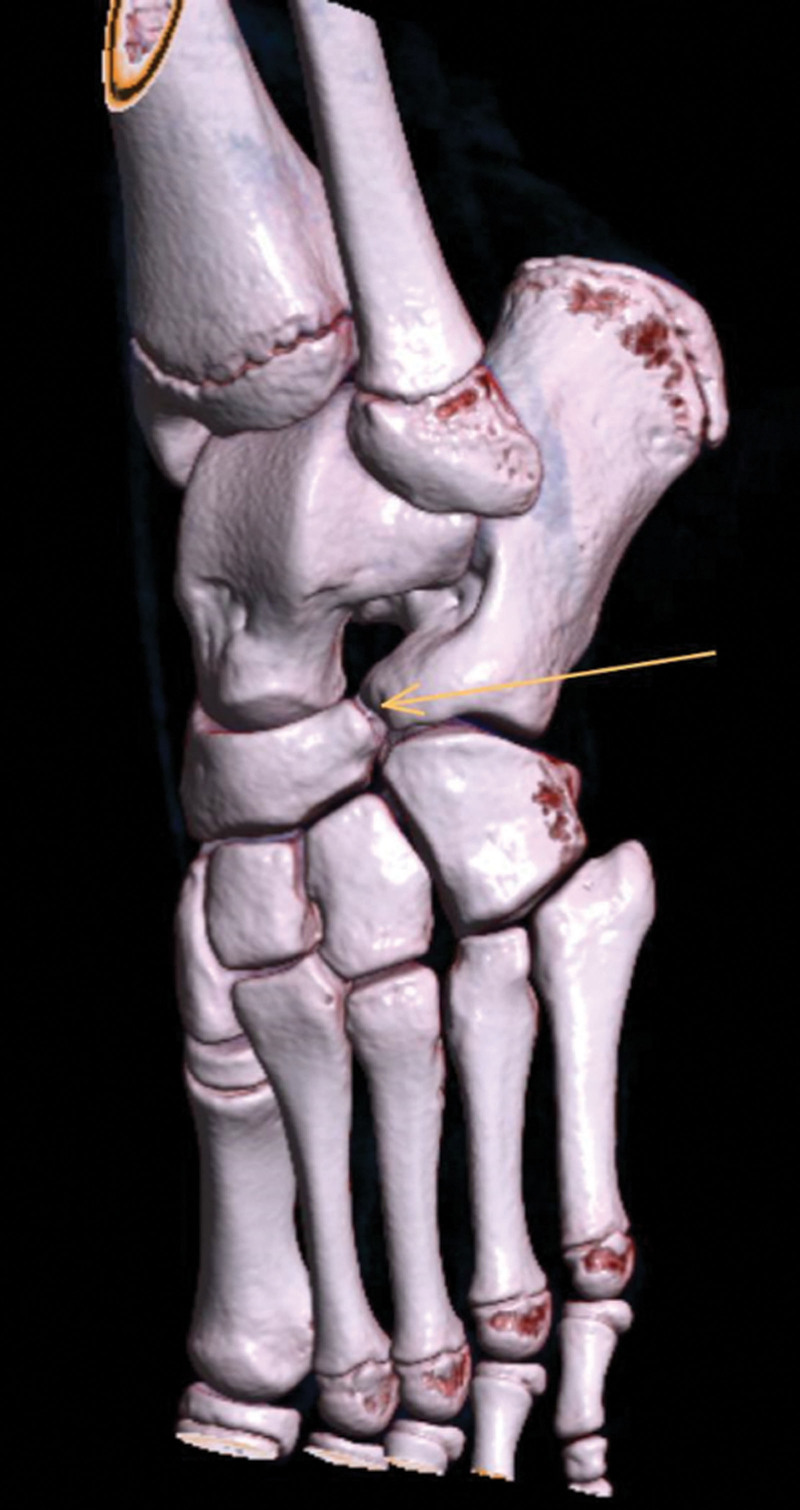
3D CT image of a calcaneonavicular coalition.

**Fig. 2:**
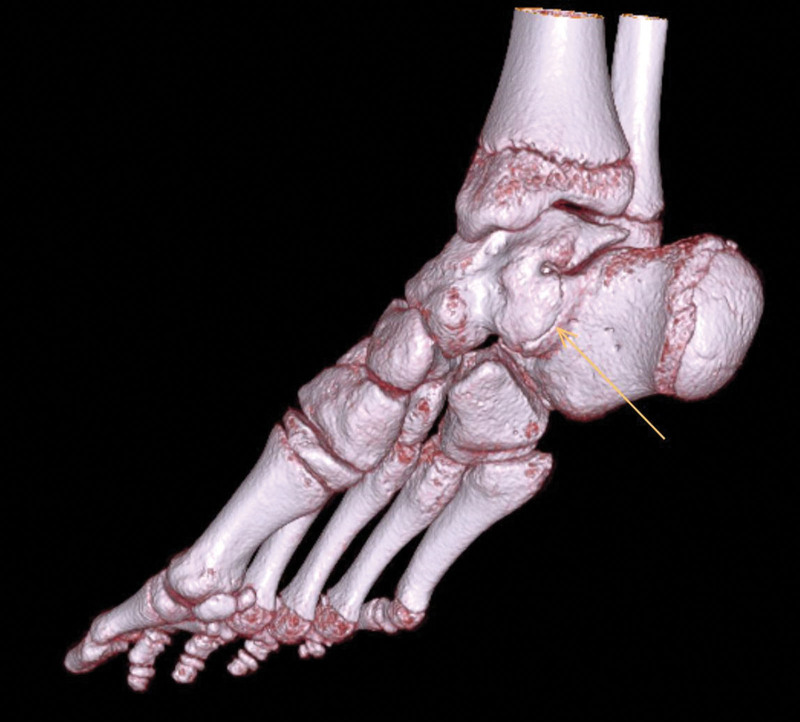
3D CT image of a talocalcaneal coalition.

Tarsal coalitions are defined as fibrous, cartilaginous, or osseous connections between two or more tarsal bones that result from a failure of differentiation and segmentation of primitive mesenchyme [1]. The calcaneonavicular (CN) joint is most commonly affected (53%), followed by the talocalcaneal (TC) joint (37%) (Figs. 1 and 2) [2]. The incidence of tarsal coalitions was recently found to be 3.5 per 100 000 children with a reported prevalence of 2 to 13% [3,4].

The insidious onset of vague and aching pain in a child between the ages of 8 and 16 years is characteristic [1]. Patients usually experience pain during physical activities, stiffness of the foot, and recurrent ankle sprain, all caused by movement restrictions in the hindfoot [1,2]. The foot may be flat with valgus of the hindfoot [2].

Radiographs can directly visualize some coalitions or show indirect but suggestive signs related to motion restriction or foot morphology [2]. The diagnosis can be confirmed with computed tomography (CT) or MRI.

Treatment is only necessary in persistently painful tarsal coalitions. Surgery is indicated if nonoperative management fails. Surgical options include resection of the coalition, deformity correction with an osteotomy, a combination of procedures, or an arthrodesis [1]. Resection is the primary surgical procedure, commonly with use of tissue interposition to prevent recurrence. Different interposition materials have been reported, including fat, bone wax and muscle or tendon [1,2,5]. It is unclear if resection alone is equally effective to resection combined with interposition. Moreover, it is unknown which interposition material provides the lowest risk of recurrence. Therefore, the aim of this study was to systematically review the literature concerning the result of tarsal coalition resection with or without different interposition materials in children.

## Materials and methods

This systematic review was performed and reported in accordance with the Preferred Reporting Items for Systematic Reviews and Meta-Analyses (PRISMA) guidelines [6].

### Literature search and study selection

Three online medical databases (PubMed, Embase, and the Cochrane Central Register of Controlled Trials) were searched on 30 April 2022 using the keywords ‘tarsal coalition’, ‘pediatric’, ‘surgery’, ‘interposition’, and their synonyms, each fitted for the specific databases, after consultation of a medical librarian. Full search details are available in Appendix I, supplemental digital content 1, http://links.lww.com/JPOB/A106. Title, abstract and full-text screening was performed by two independent reviewers to identify potentially relevant articles. Additionally, the reference lists of the included articles were manually checked to avoid missing relevant articles. The authors independently selected articles. Studies were not blinded for author, affiliation, or source. Any disagreement was resolved by discussion and consensus by the reviewers.

### Inclusion and exclusion criteria

All included articles presented original data on pediatric patients who had undergone surgical resection for tarsal coalition. Studies were included if they were written in English, German, or Dutch, had at least 12 months of mean follow-up, and reported on a minimum of five patients. Reviews, expert opinions and surgical technique articles were excluded, as well as combined procedures (e.g. additional subtalar arthroereisis). Articles that did not provide separate data for children were also excluded, just as articles that included different techniques without distinguishing their outcomes.

### Data extraction

The following parameters were extracted when available: numbers of patients and feet, sex, age, and type of coalition. Relevant outcome parameters included the duration of follow-up, pain score measured using the visual analogue scale (VAS), range of motion (ROM) of the foot in terms of dorsiflexion – plantarflexion and inversion – eversion, outcome scores as reported in the studies [e.g. American Orthopedic Foot and Ankle Society (AOFAS) ankle-hindfoot score], complications, recurrence, and revision surgery.

Three functional outcome scores were found in the studies. The AOFAS is a 100-point objective and subjective score that assesses pain, function, alignment, and joint motion. The scores are rated as excellent (90–100 points), good (80–90 points), fair (70–80 points), or poor (<70 points). The Foot and Ankle Outcome Score assesses 5 subgroups. Scores range from 0 to 100, with a score of 0 indicating the worst possible foot/ankle symptoms and 100 indicating no foot/ankle symptoms [7]. Another ankle/foot score was developed by O’Neill and Micheli, which gives 10 points in five subjective categories and 10 points in five objective categories [8]. A total of 100 points can be scored, and a score of 90–100 is regarded as excellent, 75–89 as good, 60–74 as fair, and 0–59 as poor.

### Methodological quality

The methodological quality of included studies was assessed by two independent reviewers by assigning levels of evidence as previously defined by the Centre for Evidence-Based Medicine. Any scoring differences were discussed until consensus was reached.

To assess the risk of bias, the Methodological Index for Non-Randomized Studies (MINORS) was used [9]. The MINORS is a validated and established index for evaluating the methodological quality of non-randomized studies. The index involves 12 criteria for comparative studies, of which eight criteria have been designed for noncomparative studies. These items were scored according to the set criteria: 0 (not reported), 1 (reported but inadequate), or 2 (reported and adequate). The maximum scores were 24 and 16 for comparative and cohort studies, respectively. Two reviewers independently evaluated each study according to the MINORS index and scoring differences were discussed until consensus was reached.

### Data and statistical analysis

The primary outcome measure was recurrence of the coalition (clinically and/or radiologically).

Secondary outcome measures were pain, ROM, function, revision surgery, complications, and donor site morbidity.

The outcome measures were calculated for each study by dividing them by the total number of feet. Due to the different sizes of the study populations, the average is expressed in a weighted mean: larger study populations weigh more than smaller study populations, and each patient contributes equally to the final mean. When possible and relevant, weighted mean values were also calculated according to type of coalition. Some studies compared different groups; therefore, when possible, the different groups were analyzed separately.

### Ethical approval

Ethical approval was not sought for the present study because publicly accessible documents were used as evidence. Therefore, the authors did not require to seek ethics approval before commencing this systematic review.

## Results

### Selection process

**Fig. 3:**
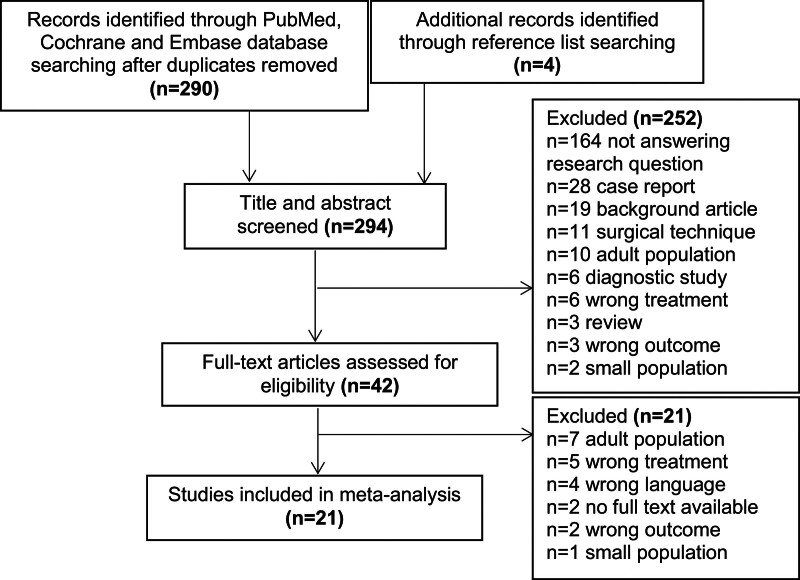
Flowchart of selection process.

The search yielded a total of 294 unique articles (flowchart presented in Fig. 3). Articles were screened for title and abstract. A total of 42 studies were selected for full-text screening and a total of 21 articles were included for data extraction. The reference lists of the included articles were manually checked to avoid missing relevant articles. An overview of the included articles and baseline characteristics is presented in Table 1. All studies had IV level of evidence.

**Table 1:** Data of the included studies

Study	Study design	Number of feet	Age (range)	Type of coalition	Follow-up (in months)	Treatment in addition to resection	Clinical outcome	Recurrence	MINORS
Hubert *et al*. 2018	Retrospective	12	12.2 (10–18)	12 TC	57	Flap of tibialis posterior tendon sheath	Mean VAS pre-op 7.3, post-op 0.3. Mean AOFAS pre-op 63, post-op 96. Mean inversion pre-op 0, post-op 30 degrees. Mean eversion pre-op 0, post-op 10 degrees.	0/12	10
Masquijo *et al*. 2017	Retrospective	56	12.2 (10–17)	56 CN	35	Fat graft (23) Bone wax (18) EDB (15)	Mean VAS pre-op 7, post-op 0.7. Mean AOFAS pre-op 53, post-op 92.	8/56	20
Gantsoundes *et al*. 2012	Retrospective	49	13.1	49 TC	43	Fat graft	Mean AOFAS post-op 90.	1/49	5
V Renthergem and De Ridder 2011	Retrospective	22	13.3 (9–16)	22 CN	58	EDB	Mean Foot and Ankle Outcome Score post-op 4.6 (range 0–5).	0/22	7
El Shazly and Abou El Ela 2011	Prospective	12	12 (11–13)	12 CN	26	Non-absorbable synthetic graft (Teflon or dacron)	Mean AOFAS pre-op 48, post-op 90.	0/12	6
Sperl *et al*. 2010	Retrospective	6	13.4 (10–15)	3 TC 1 CN 2 other (1 naviculo-cuboidal, 1 naviculo-cuneiform)	40	Deepithelialized skin flap graft	Mean AOFAS post-op 87.	0/6	5
Mubarak *et al*. 2009	Retrospective	96	12 (7–17)	96 CN	29	Fat graft	Mean VAS pre-op 6.7, post-op 0.3.	10/96	7
Westberry *et al*. 2003	Retrospective	12	12.7 (9–18)	12 TC	61	No interposition	Mean AOFAS pre-op 46, post-op 90.	0/12	6
Raikin *et al*. 1999	Retrospective	14	12 (9–16)	14 TC	51	Bone wax and half FHL tendon	Mean VAS pre-op 8, post-op 2. Mean AOFAS pre-op 54, post-op 92.	0/14	7
Dutoit 1998	Retrospective	8	11.3 (10–16)	8 TC	57	Fat graft		0/8	6
Luhmann and Schoenecker 1998	Retrospective	25	12.5(9–16)	25 TC	30	Bone wax with fat graft	Mean AOFAS post-op 82.	2/25	8
Moyes *et al*. 1994	Retrospective	17	12	17 CN	40	10 EDB 7 no interposition	9 of 10 pain free 3 of 7 pain and signs of recurrence	3/17	3
De Wilde *et al*. 1994	Retrospective	20	13 (9–15)	20 TC	12–109	No interposition	10 excellent/good, 10 fair/poor.	2/18	5
Cohen *et al*. 1993	Retrospective	13	12.3 (10–14)	13 CN	66	EDB	12 patients good or excellent result post-op.	0/13	3
Salomao *et al*. 1992	Retrospective	29	12.6 (10–17)	29 CN	25	Bone wax and fat graft	78% post-op no pain. In 75% improvement in range of motion.	n.m.	4
Gonzalez and Kumar 1990	Retrospective	75	11.2 (8–17)	75 CN	99	EDB		17/75	6
O’Neill and Micheli 1989	Retrospective	18	13 (9–17)	16 CN 1 TC 1 other (naviculo-cuboidal)	61	Bone wax with fat graft and EDB	Mean ankle-foot score pre-op 64, post-op 85.	2/16	5
Olney and Asher 1987	Retrospective	9	12.7 (10–15)	9 TC	43	Bone wax and half FHL		n.m.	5
Inglis *et al*. 1986	Retrospective	16	11.5 (10–14)	16 CN	276	No interposition	11 good/excellent	2/16	4
Chambers *et al*. 1982	Retrospective	31	12 (8–14)	31 CN	96	EDB		n.m.	5
Mitchell and Gibson 1967	Retrospective	41	11(10–14)	41 CN	72	No interposition	In 31 feet satisfactory results, in 10 unsatisfactory results.	0/41	4

CN, calcaneonavicular; EDB, extensor digitorum brevis; FHL, flexor hallucis longus; n.m., not mentioned; TC, talocalcaneal.

### Methodological quality and risk of bias

The MINORS index was applied to all selected articles and was generally low (median 7; range 3–20). The major limitations of the methodology of the selected studies were retrospective data collection and no control group. Only the comparative study of Masquijo *et al*. compared different interposition techniques including fat graft, muscle interposition, and bone wax [10].

### Overall data

In the 21 studies, a total of 436 patients (581 feet) were included (Table 1). The mean age of the patients was 12.2 years (range, 7–18). There were 153 TC, 425 CN, 2 naviculocuboidal, and 1 naviculocuneiform coalitions. Mean follow-up time was 58 months (range, 12–276). In 96 patients, only resection was performed. In the other patients, resection was followed by interposition tissue with use of a fat graft (n = 176), muscle or tendon (n = 178), other material (n = 36), or a combination of interposition techniques (n = 95). Eighteen articles reported on clinical and/or radiological recurrence of tarsal coalition, which was found in 45 of 485 feet (9%).

### Outcomes according to graft type

Recurrence of tarsal coalition was seen in 11% of the treated feet after CN coalition resection and in 4% after TC coalition resection (Table 2). Because two studies included multiple types of coalitions without enough information for differentiation, these studies were excluded from the recurrence analysis [8,11].

**Table 2:** Recurrence according to graft type

	Talocalcaneal			Calcaneonavicular		
Type of interposition	Feet (n)	Recurrence (n)	Recurrence (%)	Feet (n)	Recurrence (n)	Recurrence (%)
No interposition	29	2	6%	64	5	8%
Fat	57	1	2%	119	11	9%
Muscle/tendon	12	0	0%	135	23	17%
Bone wax				18	1	6%
Combination	39	2	8%			
Other				12	0	0%
Total	137	5	4%	348	40	11%

### No interposition

In five studies, only resection without interposition was performed (n = 96) [12–16]. Two studies described 29 patients with a TC coalition [12,13]. The mean AOFAS in the study of Westberry *et al*. was 46 preoperatively and 90 postoperatively. There were no signs of recurrence [12]. De Wilde *et al*. found recurrence in 2 patients and reported fair or poor results in the ten feet in which the coalition was greater than 50% of the subtalar joint [13].

Moyes *et al*., Inglis *et al*., and Mitchell and Gibson operated on a total of 64 patients with a CN coalition. In 20 of these patients, unsatisfactory results were found with 5 recurrences [14–16].

### Fat graft interposition

In four studies with a total of 176 feet, a fat graft was used as interposition [10,17–19]. There was a CN coalition in 119 feet [10,18] and a TC coalition in 57 feet [17,19]. In two studies including a total of 119 patients, all with CN coalitions, the weighted mean VAS pain was 6.8 preoperatively and 0.3 postoperatively [10,18]. The AOFAS was mentioned preoperatively in only one study (n = 23) [10] with a mean of 59, and postoperatively in two studies (n = 72) [10,17] with a weighted mean score of 93. Recurrence was found in 12 of 176 feet (6.8%) according to radiography or CT. In 4 patients a repeat resection was done [10,17–19].

### Muscle/tendon interposition

In seven studies, interposition with muscle/tendon was used for 166 CN coalitions and 12 TC coalitions [10,14,20–24]. The extensor digitorum brevis (EDB) was the most commonly used interposition material (Table 1). Two studies mentioned the VAS pain and AOFAS: Hubert *et al*. included 12 TC coalitions and Masquijo *et al*. 15 CN coalitions with a weighted mean preoperative VAS of 7.1 and postoperative score of 1.1 [10,20]. The mean weighted AOFAS scores were 53.8 preoperatively and 83.3 postoperatively [10,20]. Moyes *et al*. mentioned that 9 of 10 patients were pain-free postoperatively [14]. Only the study of Hubert described the ROM; they found no inversion or eversion preoperatively, and an increase in ROM of 30 degrees of inversion and 10 degrees of eversion [20].

Recurrence was mentioned in all studies except for the study of Chambers *et al*. [10,14,20–24] In a total of 147 feet, partial reformation was found in 17 feet [23], and complete recurrence occurred in 6 feet (total 16.8%) [10]. Two of these patients had repeat resection [10]. In the other four studies, no recurrences were seen (Hubert, Van Renthergem, Cohen, Moyes) [14,20–22].

### Bone wax

Bone wax was used in one study including 18 feet [10]. The preoperative mean VAS was 7, and the postoperative VAS was 0 in all patients. The mean AOFAS was 50 preoperatively and 98 postoperatively. In one patient recurrence was found (5.6%).

### Combination

A combination of interposition techniques was done in five studies including a total of 94 feet [8,25–28]. One study used bone wax with tendon (n = 14) [25], three used bone wax and fat graft (n = 63) [26–28], and one used bone wax with tendon and fat graft (n = 18) [8]. VAS pain in one study decreased from a mean of 8.1 preoperatively to 1.6 postoperatively [25]. The AOFAS increased from a mean of 54 to 92 in the study of Raikin *et al*. and was 82 postoperatively in the study of Luhmann and Schoenecker [25,28], Salomao *et al*. mentioned that 78% of patients had no pain postoperatively [26]. O’Neill *et al*. used an alternative ankle-foot score and found an increase in this score from 64 preoperatively to 85 postoperatively [8]. Three studies reported on recurrence (n = 55), which was found in four feet. Three of these feet had additional surgeries [8,25,28].

### Other

Two studies used other interposition material: El Shazly and Abou El Ela used non-absorbable synthetic graft (Teflon or Dacron) in 12 patients, and Sperl *et al*. used deepithelialized skin flap in 6 patients [11,29]. The AOFAS was mentioned in both studies postoperatively and in El Shazly and Abou El Ela also preoperatively. The preoperative AOFAS was 48 and the mean weighted postoperative score was 89. Both studies found no recurrence [11,29].

## Discussion

To our knowledge, this is the first systematic review evaluating different interposition techniques after surgical resection of tarsal coalition in children. This systematic review shows that in the treatment of both TC and CN coalitions, coalition resection combined with various interposition techniques generally results in low recurrence rates and improvement of complaints in children. Interestingly, less recurrence following TC coalition resection was seen compared to CN coalition resection. However, evaluating the outcomes of different interposition techniques for CN and TC coalition resection was limited due to the heterogeneity of data, different outcome measures and variable follow-up durations.

Each interposition technique has advantages and disadvantages. The EDB was the most commonly used graft as muscle interposition, possibly because it is available through the incision on the foot in case of a CN coalition. However, there was a risk of recurrence of 17% (Table 2), which might be explained by the completeness of interposition due to length of the tendon/muscle. Mubarak *et al*. showed, in a cadaveric study, that the EDB only filled 64% of the gap after CN coalition resection [18]. The changed position of the EDB can also result in bony prominence on the lateral aspect of the foot which can cause discomfort when wearing shoes [5,18]. Tendon interposition (a split of the flexor hallucis longus or tibialis posterior tendon) can also fill the gap and prevent recurrence, although limitation in active motion of the foot or great toe, rupture of the tendon, or hallux deformity have been described [20].

A fat graft is another frequently used interposition tissue. Similar to muscle/tendon, autologous fat graft has the advantage of being a biologic method to fill the gap [5]. It can be harvested from the operation site or a free fat graft can be used. In the latter case, a separate incision is needed, but the advantage might be to harvest a bigger and more robust fat graft compared to fat graft from the operation site. In the present review, a recurrence rate of 7% was found with the use of fat interposition. Four studies used different fat graft donor sites [10,17–19].

Bone wax has been used as an alternative interposition material. Bone wax consists of sterilized white-bleached honeybees wax blended with a softening agent. It has the advantage of avoiding an accessory incision but once placed is not reabsorbed. Bone wax is known to interfere with bone healing and osteogenesis, which is advantageous in the surgical management of coalitions. However, bone wax has been shown to induce chronic inflammation, and to reduce bacterial clearance in cancellous bone, potentially increasing the risk of infection, although this was not shown [10]. The advantage of synthetic material is also avoidance of an additional incision and, if placed properly, no bone healing. However, adverse tissue reactions have been described and the additional costs should be counted.

De Wilde *et al*. and Comfort *et al*. made preoperative CT scans in patients with a TC coalition and both found a relationship between the extent of the coalition and the chance of unsatisfactory results [13,30]. Comfort concluded that coalitions involving one-third or less of the total joint surface had an almost 80% likelihood of good or excellent result. If the coalition occupied more than one-third of total joint surface, 75% of their results were poor or fair [30]. De Wilde found an unsatisfactory result in feet in which preoperative CT showed a relative coalition area of greater than 50%, heel valgus of more than 16 degrees, narrowing of the posterior TC joint and impingement of the lateral talar process on the calcaneum. Talar beaking on preoperative radiographs did not correlate with outcome [13]. Therefore, it might be of interest to obtain preoperative CT scans in patients with TC coalitions to determine the extent of the coalition and to estimate the chance of success.

One of the strengths of this review is the systematic search method to identify relevant articles for this subject, with methodology according to PRISMA guidelines. In addition, this review is the first systematic review reporting on treatment methods for tarsal coalitions in children. In that light, the results of the present review are useful for pediatric orthopedic surgeons treating children with coalitions.

There are also several limitations. Although it was possible to include 21 studies with a total of 581 feet, almost all studies were retrospective, had low MINOR scores and included small numbers of patients. The study of Masquijo *et al*. was the only comparative study with different interposition techniques (fat graft, muscle interposition, and bone wax) and found more recurrence in the group with muscle interposition [10]. Unfortunately, no clinical assessment was done on any of these patients. The authors only looked at any evidence of bony reformation which they defined as a recurrence even if the bony reformation was minimal or partial. There is a big difference between a small amount of bony reformation about the site of a coalition excision and a true recurrence of the coalition. Second, some studies did not (completely) report all relevant outcomes, including the recurrence rates, pain, ROM, and function, while objective clinical and radiological outcome with adequate follow-up are essential to appreciate the results of the studies reliably. Therefore, possible differences between the interposition techniques in the different coalitions are not known. It was not possible to perform a meta-analysis on these data because of the diversity in treatment options and coalitions, the heterogeneous nature of the studies, and the disproportionate distribution of no interposition versus the different interposition techniques.

The variation in practice highlights the uncertainty that remains in the orthopedic community about the best methods to treat this condition. It seems that fat graft or bone wax interposition achieve the least recurrences. Future studies should include patients prospectively and compare different interposition techniques (ideally in a randomized controlled way), report adequate pre- and postoperative data about pain, function, ROM, and radiological outcome, with sufficient follow-up times.

## Acknowledgements

Arnela Haagmans-Suman is kindly acknowledged for statistical advice.

### Conflicts of interest

There are no conflicts of interest.

## Supplementary Material
