# Supplementary material for: Comparison of different interposition techniques after surgical resection of tarsal coalitions in children: a systematic review
Source: J Pediatr Orthop B. 2024 Apr 9;34(1):1–8. doi: 10.1097/BPB.0000000000001177 (PMC11594538; doi:10.1097/BPB.0000000000001177)
Supplement: Supplementary file 1 [file jpob-34-01-s001.pdf]

Appendix I

Search for PubMed (comparable searches for Embase and Cochrane):

(("Tarsal Coalition"[Mesh] OR "Foot Deformities, congenital"[MESH:noExp] OR "Talonavicular coalition"[supplementary concept]) OR ((tarsal[tiab] OR talus[tiab] OR talonavicular[tiab] OR talocalcaneal[tiab] OR calcaneonavicular[tiab] OR "Talus"[MESH] OR "Tarsal bones"[Mesh]) AND (Coalition[tiab] OR coalitions[tiab] OR synostosis[tiab] OR synostoses[tiab] OR syndesmosis[tiab] OR synchondrosis[tiab] OR synchondroses[tiab] OR "Synostosis"[Mesh:NoExp] OR bar[tiab]))) AND ("General surgery"[MeSH] OR "surgical procedures, operative"[MeSH] OR surgery[tiab] OR surgical procedures[tiab] OR resection[tiab] OR surgical resection[tiab] OR open surgery[tiab] OR endoscopic surgery[tiab]) AND (Interposition[tiab] OR soft tissue[tiab] OR muscle[tiab] OR fat[tiab] OR bone wax[tiab] OR subtalar[tiab])
